# Geometry–Dependent Magnetoelectric and Exchange Bias Effects of the Nano L–T Mode Bar Structure Magnetoelectric Sensor

**DOI:** 10.3390/mi14020360

**Published:** 2023-01-31

**Authors:** Treetep Saengow, Rardchawadee Silapunt

**Affiliations:** Electronic and Telecommunication Engineering, King Mongkut’s University of Technology Thonburi, 126 Prachauthit Rd., Thung Kru, Bangkok 10140, Thailand

**Keywords:** exchange bias effect, magnetoelectric coefficient, magnetoelectric effect, magnetostrictive, multiferroic

## Abstract

The geometry–dependent magnetoelectric (ME) and exchange bias (EB) effects of the nano ME sensor were investigated. The sensor consisted of the Longitudinal–Transverse (L–T) mode bi–layer bar structure comprising the ferromagnetic (FM) and ferroelectric (FE) materials and the anti–ferromagnetic (AFM) material. The bi–layer ME coefficient was derived from constitutive equations and Newton’s second law. The trade–off between peak ME coefficient and optimal thickness ratio was realized. At the frequency × structure length = 0.1 and 1200, minimum and maximum peak ME coefficients of the Terfenol–D/PZT bi-layer were around 1756 and 5617 mV/Oe·cm, respectively, with 0.43 and 0.19 optimal thickness ratios, respectively. Unfortunately, the bi-layer could not distinguish the opposite magnetic field directions due to their similar output voltages. PtMn and Cr_2_O_3_, the AFM, were introduced to produce the EB effect. The simulation results showed the exchange field starting at a minimum PtMn thickness of 6 nm. Nevertheless, Cr_2_O_3_ did not induce the exchange field due to its low anisotropy constant. The tri–layer ME sensor consisting of PZT (4.22 nm)/Terfenol–D (18 nm)/PtMn (6 nm) was demonstrated in sensing 2 Tbit/in^2^ magnetic bits. The average exchange field of 5100 Oe produced the output voltage difference of 12.96 mV, sufficient for most nanoscale magnetic sensing applications.

## 1. Introduction

The magnetoelectric (ME) effect is a phenomenon of magnetic and electric coupling observed in magnetoelectric materials and multiferroic (MF) composites, which consist of ferroelectric (FE) and ferromagnetic (FM) materials. Initially, the ME effect was studied slowly due to a lack of approaches to find its essence and benefits [1]. The first ME effect was postulated in 1894, but not until 1960 was it observed experimentally [2]. However, after 2000, recognition of the usefulness of magnetic sensing applications led to extensive studies of the ME effect [2]. The ME materials and multiferroic composites can convert the energy between the magnetic and electric fields through the magnetostrictive and piezoelectric effects [3,4,5]. The magnetic and electric energies are placed in the form of magnetization and polarization, respectively. The mechanical stress and strain associated with the magnetostrictive and piezoelectric effects are key to coupling FM and FE materials to produce the ME effect. Generally, the ME effect’s efficiency is measured as the ratio between the electric field and the applied magnetic field, known as the ME coefficient [6,7]. The induction of the electric field by the magnetic field is called the direct ME coupling effect, while the induction of the magnetic field by the electric field is called the converse ME coupling effect [3,4,5]. Due to their simpler fabrication, the MF composites are mostly formed in the heterostructure, consisting of alternate layers of FE and FM materials, bi- or tri-layer. It was discovered that MF composites possessing large ME effects have excellent magnetic sensing capabilities. Various research and studies have thus focused on producing large ME effects, such as pairing different MF materials, adding some compounds, modifying fabricating techniques and procedures, etc. [4,5,8,9]. It has also been found that the ME effect’s properties are highly geometry–dependent and that the operating frequency is another significant factor altering the ME effect’s sensing capability [6,7,8,9,10,11,12,13]. Several studies have also focused on developing mathematical models of the ME coefficient to explain, understand, and improve the ME effect for sensing applications [6,7,11,12,13].

The heterostructure MF composites are basically categorized in four operation mode–structures: the longitudinal–transverse (L–T) and transverse–transverse (T–T) modes of the bar and plate structures. The long and rigorous derivation proves that the bar structure of an MF composite, regardless of mode of operation, is always smaller than the plate structure [12]. More specifically, the ME coefficient of the L–T mode bar structure is always higher than the T–T mode [12,14]. Therefore, the L–T mode bar structure is a preferred choice in sensing applications as it offers higher flexibility for structure and dimension design that is usually a large constraint when it comes to sensor fabrication. However, the MF composites alone are insufficient to distinguish between media fields possessing the same magnitude but opposite directions, i.e., upward vs. downward, since the resulting electric field strengths and thus the voltage outputs will be the same value.

The exchange bias (EB) effect is key to eliminating this problem and promoting ME effect enhancement [15,16,17]. The EB effect is formed by coupling the FM and anti-ferromagnetic (AFM) materials [15,17,18]. The effect is considered geometry-dependent for some AFM materials. When the exchange bias occurs, the FM is biased and its hysteresis loop is shifted over to one side, resulting in clearly different applied field strengths for the fields in the opposite directions. This exchange bias effect when coupling to the FE layer will help produce different electric field strengths across the FE layer. Therefore, the voltage output will also differ following the media field directions.

In this paper, we aimed to understand the dependence of ME characteristics and the EB effect on the geometry of the nano ME sensor. The ME characteristics were investigated via the ME coefficient of the nano bi–layer L–T bar structure MF composites, which was evaluated through well-developed mathematical models. The ME coefficient profiles were demonstrated using the bi-layer of Lead Zirconate Titanate (PZT) as the FE material and Terbium Dysprosium Iron (Terfenol–D), a widely used FM material possessing a strong magnetostrictive coefficient, as the FM material [4,5,19].

Then, the EB effect was investigated using Mumax^3^, a magnetic simulation program. We then assembled and compared two FM/AFM nano bi-layers: The first is the Terfenol–D (FM) and Platinum Manganese (PtMn) and the second is Terfenol–D (FM) and Chromium tri-oxide Cr_2_O_3_ (AFM).

**Figure 1:**
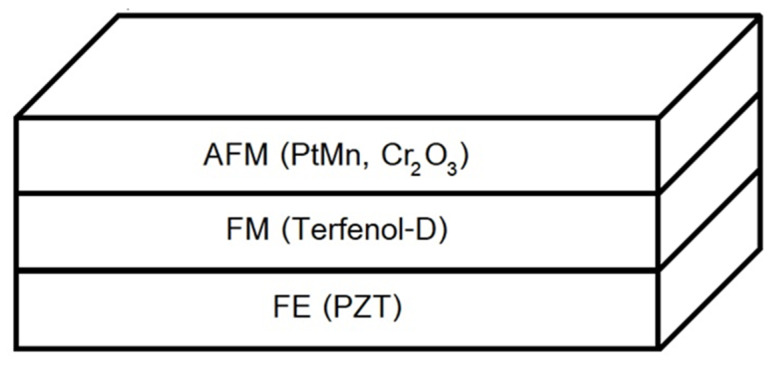
The tri–layer sensor structure consists of FE, FM, and AFM.

We demonstrated the EB effect through the hysteresis loops resulting from the AFM materials and their thicknesses. Finally, the demonstration of the nano tri-layer AFM/FM/FE structure, shown in Figure 1 as a nano read head sensor in the modern hard disk drive (HDD), is presented. The ME effect and EB effect were considered together, and the results were assessed for the possibility of applying the ME sensor in nanoscale magnetic sensing applications.

## 2. Mathematical Model Development of the ME Coefficient for the L–T Mode Bi–Layer Bar Structure

**Figure 2:**
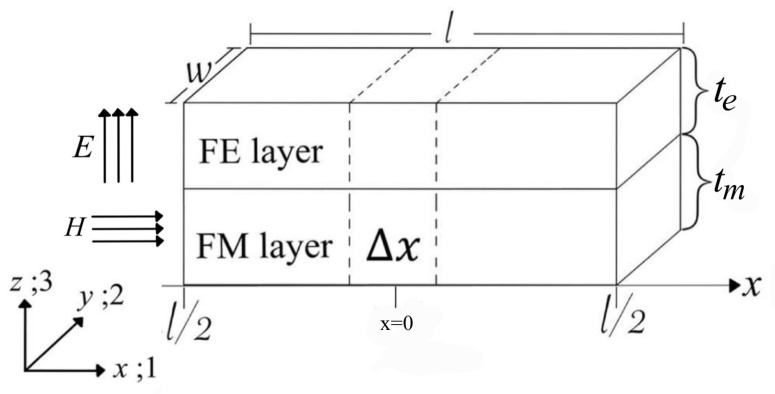
The bi–layer L–T mode bar structure.

We constructed the bi–layer L–T mode bar structure of the nano MF composites in the shape of a laminate structure. The structure consisted of FM and FE layers with length (*l*), width (*w*), and total thickness (*t*), as shown in Figure 2. Note that the design condition was *l* >> *w*, *t* [11,20]. We considered the magnetostriction induced by the applied magnetic field in the longitudinal direction. This magnetostrictive effect was coupled to the FE layer, resulting in an electric field induced in the transverse direction.

The magnetostrictive constitutive equation and piezoelectric constitutive equation are linked via mechanical stress and strain and used Newton’s second law of motion [21]. These stress and strain occurred in the longitudinal direction. We assumed strong coupling between FM and FE layers; thus, the elastic displacements, ξ in both layers were equal—$\xi_{1}^{m}=\xi_{1}^{e}=\xi_{1}$. Since the forces acting on FM and FE were in the same direction, the total force became

(1) $$\frac{d\sigma_{1}^{e}}{dx}t_{e}+\frac{d\sigma_{1}^{m}}{dx}t_{m}=\left(\rho_{e}t_{e}+\rho_{m}t_{m}\right)\frac{d^{2}\xi_{1}}{dt^{2}},$$

where σ  is the stress [N/m^2^], ρ  is the material density [kg/m^3^], superscript ‘*m*’ refers to a ferromagnetic material, superscript ‘*e*’ refers to a ferroelectric material, and subscript ‘1′ refers to the longitudinal direction.

Given the total thickness t as $t=t_{e}+t_{m}$ and the thickness ratio η as $\eta =\frac{t_{e}}{t}$, it could be shown that $t_{e}=\eta t$ and $t_{m}=\left(1-\eta \right)t$. Rewriting Equation (1) yielded Newton’s second law of motion as

(2) $$\eta \frac{d\sigma_{1}^{e}}{dx}+\left(1-\eta \right)\frac{d\sigma_{1}^{m}}{dx}=\left[\eta \rho_{e}+\left(1-\eta \right)\rho_{m}\right]\frac{d^{2}\xi_{1}}{dt^{2}}.$$

The stress form of the FM and FE layers could be rewritten as

(3) $$\sigma_{1}^{m}=\frac{\chi_{1}^{m}}{s_{11}^{m}}-\frac{d_{11}^{m}}{s_{11}^{m}}H_{1}$$

(4) $$\sigma_{1}^{e}=\frac{\chi_{1}^{e}}{s_{11}^{e}}-\frac{d_{31}^{e}}{s_{11}^{e}}E_{3},$$

where χ is the strain [dimensionless], *s* is the elastic compliance [m^2^/N], $d^{m}$ is the magnetostrictive constant [m/A], $d^{e}$ is the piezoelectric constant [m/V], and ‘3′ refers to the thickness direction. Taking the derivative of Equations (3) and (4) with respect to x direction yielded

(5) $$\frac{\partial \sigma_{1}^{m}}{\partial x}=\frac{1}{s_{11}^{m}}\frac{\partial \chi_{1}^{m}}{\partial x}$$

(6) $$\frac{\partial \sigma_{1}^{e}}{\partial x}=\frac{1}{s_{11}^{e}}\frac{\partial \chi_{1}^{e}}{\partial x}.$$

Referred to the definition of strain, $\chi =\frac{\Delta L}{L_{ο}}$, since the FM and FE layers were rigidly strong coupling; therefore, $\chi_{1}^{m}=\chi_{1}^{e}=\chi_{1}$; thus, Equations (5) and (6) were equated such that

(7) $$\frac{\partial \chi_{1}^{m}}{\partial x}=\frac{\partial \chi_{1}^{e}}{\partial x}=\frac{\partial \chi_{1}}{\partial x}=\frac{\partial^{2}\xi_{1}}{\partial x^{2}}.$$

Combining the Equations (5)–(7) yielded

(8) $$\frac{\partial \sigma_{1}^{m}}{\partial x}=\frac{1}{s_{11}^{m}}\frac{\partial^{2}\xi_{1}}{\partial x^{2}}$$

(9) $$\frac{\partial \sigma_{1}^{e}}{\partial x}=\frac{1}{s_{11}^{e}}\frac{\partial^{2}\xi_{1}}{\partial x^{2}}.$$

Substituting Equations (8) and (9) into Equation (2) yielded

(10) $$\left[\frac{\eta s_{11}^{m}+\left(1-\eta \right)s_{11}^{e}}{s_{11}^{m}s_{11}^{e}}\right]\frac{\partial^{2}\xi_{1}}{\partial x^{2}}=\left[\eta \rho_{e}+\left(1-\eta \right)\rho_{m}\right]\frac{d^{2}\xi_{1}}{dt^{2}}.$$

Rewriting Equation (10) in the frequency domain yielded

(11) $$\left[\frac{\eta s_{11}^{m}+\left(1-\eta \right)s_{11}^{e}}{s_{11}^{m}s_{11}^{e}}\right]\frac{\partial^{2}\xi_{1}}{\partial x^{2}}+\left[\eta \rho_{e}+\left(1-\eta \right)\rho_{m}\right]\omega^{2}\xi_{1}=0,$$

where *ω* is frequency.

Referred to the second-order linear differential equation form, Equation (11) was rewritten as

(12) $$\frac{d^{2}\xi_{1}}{dx^{2}}+k^{2}\xi_{1}=0,$$

where $k^{2}=\omega^{2}\frac{\left[\eta \rho_{e}+\left(1-\eta \right)\rho_{m}\right]s_{11}^{m}s_{11}^{e}}{\eta s_{11}^{m}+\left(1-\eta \right)s_{11}^{e}}$ and the second-order linear differential equation gave a solution in a sinusoidal form.

Combining Equations (3) and (4) and the definition of strain yielded

(13) $$\sigma_{1}^{m}=\frac{1}{s_{11}^{m}}\left(\frac{d\xi_{1}}{dx}-d_{11}^{m}H_{1}\right)$$

(14) $$\sigma_{1}^{e}=\frac{1}{s_{11}^{e}}\left(\frac{d\xi_{1}}{dx}-d_{31}^{e}E_{3}\right).$$

The second–order linear differential equation was applied into Equations (13) and (14) and yielded the stresses as

(15) $$\sigma_{1}^{m}=\frac{1}{s_{11}^{m}}\left[k\left(B\cos\left(kx\right)-A\sin\left(kx\right)\right)-d_{11}^{m}H_{1}\right]$$

(16) $$\sigma_{1}^{e}=\frac{1}{s_{11}^{e}}\left[k\left(B\cos\left(kx\right)-A\sin\left(kx\right)\right)-d_{31}^{e}E_{3}\right].$$

The boundary condition was then applied to determine the constant *A* and *B* at $x=\pm \frac{l}{2}$. Assuming the surrounding was free space, the external force and stress were then zero. Equation (2) became

(17) $$\eta \sigma_{1}^{e}\left(\frac{l}{2}\right)+\left(1-\eta \right)\sigma_{1}^{m}\left(\frac{l}{2}\right)=0$$

(18) $$\eta \sigma_{1}^{e}\left(-\frac{l}{2}\right)+\left(1-\eta \right)\sigma_{1}^{m}\left(-\frac{l}{2}\right)=0.$$

Substituting Equations (15) and (16) into Equations (17) and (18), respectively, and performing subtraction yielded

(19) $$A=0,\;B=\frac{\eta s_{11}^{m}d_{31}^{e}E_{3}+\left(1-\eta \right)s_{11}^{e}d_{11}^{m}H_{1}}{k\left[\eta s_{11}^{m}+\left(1-\eta \right)s_{11}^{e}\right]\cos\left(\frac{kl}{2}\right)}.$$

In order to calculate the ME coefficient, the evaluation of electric field across the FE layer was necessary. The piezoelectric constitutive equation was applied and rewritten as

(20) $$D_{3}\left(x\right)=d_{31}^{e}\sigma_{1}^{e}\left(x\right)+\varepsilon_{33}E_{3},$$

where ε  is permittivity [F/m].

The electric field was found by determining the current flow through the FE layer and zeroing it out under the open circuit condition. The relations between the current and electric charge and the electric displacement and electric charge were rewritten in the form of the current flowing through the sensor as

(21) $$I_{3}=\int \frac{dD_{3}}{dt}dA,$$

where dA=wdx the differential area through which the current flows.

Substituting Equation (20) and transferring Equation (21) to the frequency domain yielded

(22) $$I_{3}=i\omega w\int_{-\frac{l}{2}}^{\frac{l}{2}}\left(d_{31}^{e}\sigma_{1}^{e}\left(x\right)+\varepsilon_{33}E_{3}\right)dx=i\omega w\left[d_{31}^{e}\int_{-\frac{l}{2}}^{\frac{l}{2}}\sigma_{1}^{e}\left(x\right)dx+\int_{-\frac{l}{2}}^{\frac{l}{2}}\varepsilon_{33}E_{3}dx\right].$$

Now, introducing the constant *A* and *B* into Equation (15) yielded

(23) $$\sigma_{1}^{e}\left(x\right)=\frac{1}{s_{11}^{e}}\left[\frac{\eta s_{11}^{m}d_{31}^{e}E_{3}+\left(1-\eta \right)s_{11}^{e}d_{11}^{m}H_{1}}{\left[\eta s_{11}^{m}+\left(1-\eta \right)s_{11}^{e}\right]\cos\left(\frac{kl}{2}\right)}\cos\left(kx\right)-d_{31}^{e}E_{3}\right].$$

Under the open circuit condition, the current across the FE layer is equal to zero. Equation (23) was substituted into Equation (22), which was then simply reduced to

(24) $$\left[\frac{d_{31}^{e}{}^{2}}{s_{11}^{e}}-\frac{\eta s_{11}^{m}d_{31}^{e}{}^{2}}{\left[\eta s_{11}^{m}+\left(1-\eta \right)s_{11}^{e}\right]\frac{kl}{2}}\tan\left(\frac{kl}{2}\right)-\varepsilon_{33}\right]E_{3}=\frac{\left(1-\eta \right)s_{11}^{e}d_{11}^{m}}{\left[\eta s_{11}^{m}+\left(1-\eta \right)s_{11}^{e}\right]\frac{kl}{2}}\tan\left(\frac{kl}{2}\right)H_{1}$$

Rearranging Equation (24), the ME coefficient, which is the ratio between the induced electric field and the applied magnetic field, was expressed as

(25) $$\alpha_{31}^{bar}=\frac{E_{3}}{H_{1}}=\frac{\left(1-\eta \right)s_{11}^{e}d_{31}^{e}d_{11}^{m}\frac{\tan\left(\frac{kl}{2}\right)}{\frac{kl}{2}}}{d_{31}^{e}{}^{2}\left[\eta s_{11}^{m}\left(1-\frac{\tan\left(\frac{kl}{2}\right)}{\frac{kl}{2}}\right)+\left(1-\eta \right)s_{11}^{e}\right]-\varepsilon_{33}s_{11}^{e}\left[\eta s_{11}^{m}+\left(1-\eta \right)s_{11}^{e}\right]}$$

The ME coefficient was indeed a function of dimensions including length, thickness, and frequency of operation. Therefore, the optimal ME coefficient at a certain frequency could be achieved with an optimal structure dimension.

To demonstrate the geometry–dependent ME characteristics, Terfenol–D and PZT, which have high magnetostrictive and piezoelectric constants, respectively, were selected. The material properties are shown in Table 1.

## 3. Investigation of the Exchange Bias Effect in the AFM/FM Bi-Layer

For the magnetic sensing application, the expected output of the ME sensor is certainly the voltage signal with a measurable magnitude. Since, in our context, the dimension of the bi–layer was in the nanoscale, the electric field across the bi–layer structure was thus assumed uniform, and the voltage output was simply calculated by the product of ME coefficient, applied magnetic field, and FE thickness as

(26) $$V_{out}=\alpha_{31}^{bar}H_{1}t_{e}.$$

**Figure 3:**
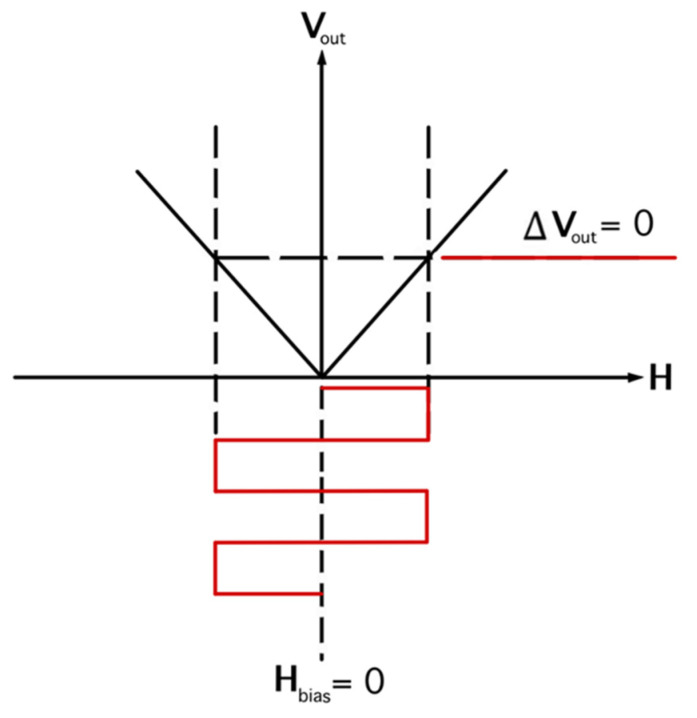
The voltage output as a function of applied magnetic field when H_bias_ = 0.

Unfortunately, the bi–layer of the ME sensor produced a similar output voltage for the same magnitude of magnetic field applied in the opposite directions, as shown in Figure 3.

**Figure 4:**
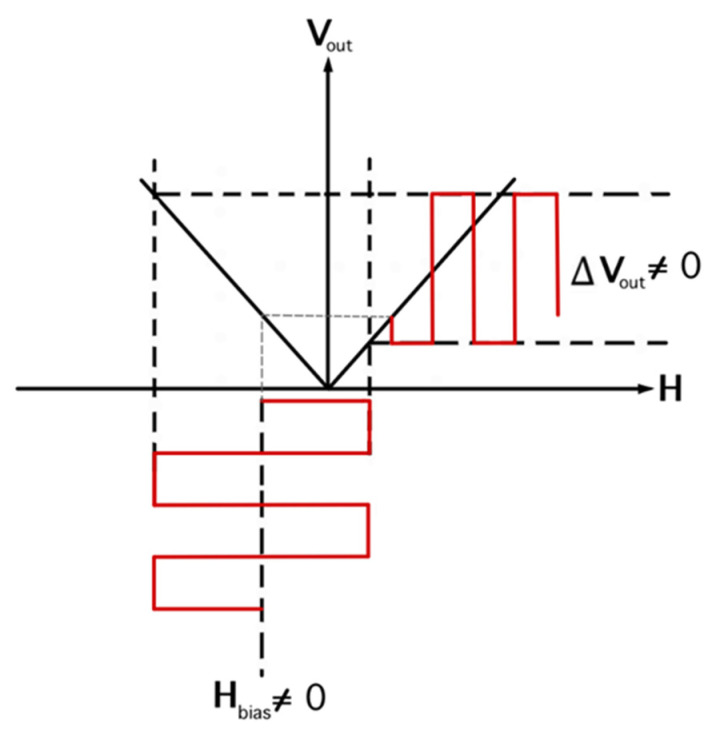
The voltage output as a function of the applied magnetic field when H_bias_ ≠ 0.

The optimal way to solve this problem is to add the constant bias field to the applied magnetic field, resulting in field biasing in one direction. The effect of the bias field is magnetic profile shifting in the FM layer, which results in different output voltages when the applied field is switched to the opposite direction, as demonstrated in Figure 4. This phenomenon is known as the exchange bias effect, which can be observed from the AFM/FM bi–layer. The strength of the EB effect significantly depends on type and geometry. In this research, we compared the EB effect of two AFM materials, PtMn and Cr_2_O_3_, at different thicknesses.

The Mumax^3^ simulation program was used to investigate the exchange bias effect. The simulator can simulate the AFM/FM bi–layer and display the FM hysteresis loops and magnetic moment variation induced in each layer by the applied magnetic field. Terfenol–D was used as the FM material coupling to two selections of AFM materials, PtMn and Cr_2_O_3_, for comparison. Note that, the two AFM materials were selected due to their excellent adhesion to Terfenol–D. The cell size was equal 3 × 3 × 3 nm^3^. The size of Terfenol–D was set up with a width and thickness equal to 36 and 18 nm, respectively. The AFM materials were also set up with the same width and thickness was varied from 3 to 18 nm. The structure length was later determined after obtaining the optimal value of the product of operational frequency and structure length (*fl*). The room temperature of 300 K, which is a typical operating temperature of the read sensor in HDD, was assumed. We configured the exchange interaction to start from a uniform and finite magnetization in AFM and FM layers [24] by setting uncompensated spins at the AFM interface to imitate the AFM spins resulted from the field cooling process. The key attributing material properties such as saturation magnetization, exchange stiffness, anisotropy constant, and surface energy density were defined in the structure setup. Their properties are shown in Table 2.

## 4. Results and Discussion

### 4.1. ME Characteristics of Terfenol–D and PZT Nano L–T Mode Bi-Layer Bar Structure

**Figure 5:**
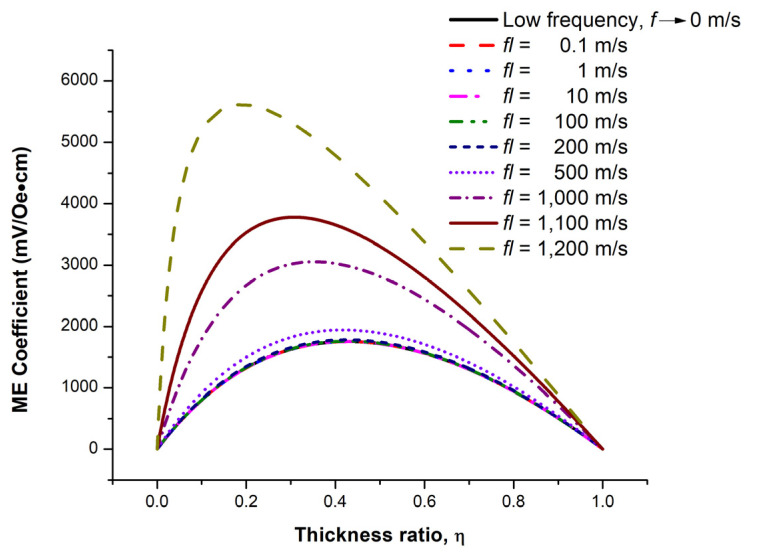
The ME coefficient as a function of thickness ratios for *fl* = 0.1–1200 m/s [30].

From the ME coefficient mathematical model development, we found that the ME coefficient was influenced by both structure dimension and frequency. For the proposed structure, we found the maximum value of *fl* at around 1228 m/s with η = 0.19 limit. The range of *fl* was then selected between 0.1 and 1200 m/s. Figure 5 shows the ME coefficient as a function of thickness ratios at *fl* = 0.1–1200, and *f* → 0 m/s. All ME coefficient traces exhibit a bell-like shape with the highest ME coefficient at *fl* = 1200 m/s.

**Figure 6:**
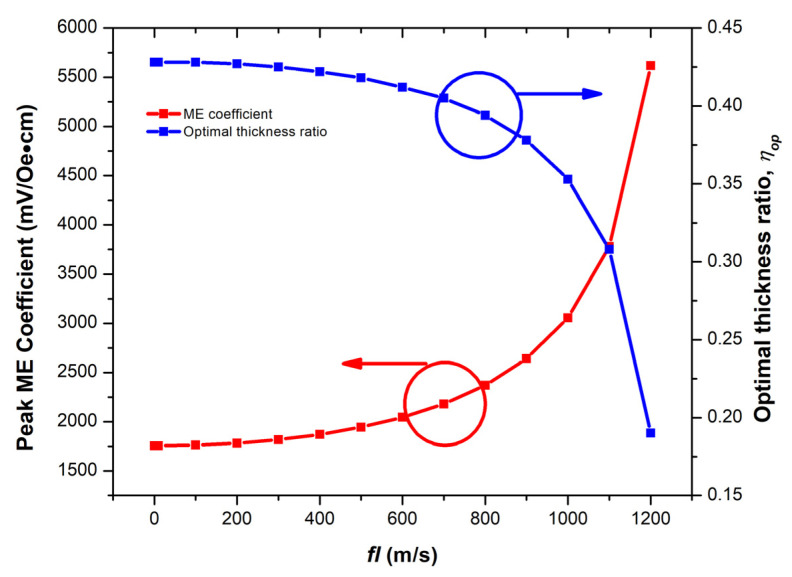
The peak ME coefficient and optimal thickness ratio as a function of *fl* [30].

From Figure 5, the thickness ratio that provides the peak ME coefficient at each *fl* was defined as the optimal thickness ratio. The plot of the optimal thickness ratio and corresponding peak ME coefficient as a function of *fl* is shown in Figure 6. The exponential trend observed from both traces can be explained by the dependence on the tan(*kl*/2)/(*kl*/2) term in Equation (25). This finding indicates the trade-off of the ME coefficient and thickness ratio in the ME sensor design. The minimum and maximum peak ME coefficients at *fl* = 0.1 and 1200 m/s, respectively, were around 1756 and 5617 mV/Oe·cm with η = 0.43 and 0.19, respectively. The results were compared with the experimental results obtained from the bulk or millimeter-scale Terfenol–D–based ME sensors reported in [8,9]. Although our proposed structure is the thin film type, similar ME characteristics were observed such as the resonant frequency and peak ME coefficient dependence significantly on the structure dimension and comparable values of the peak ME coefficient when considering the same structure dimensions.

### 4.2. The Exchange Bias Effect in the PtMn/Terfenol–D and PtMn-Cr_2_O_3_ Bi-Layers

**Figure 7:**
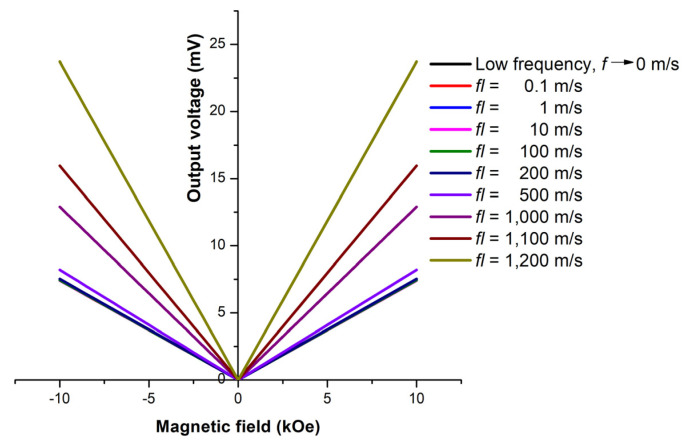
The readback voltage as a function of external magnetic field for *fl* = 0.1–1200 m/s.

Figure 7 shows the plot of voltage outputs for different *fl* of the ME bi-layer sensor. The FM thickness of the bi-layer was setup to 18 nm, equivalent with the bit length at 2 Tb/in^2^ areal density, the projected areal density of the modern hard disk drive (HDD) [31]. The symmetrical traces around the y axis indicates identical output voltages for the oppositely applied magnetic field directions. Therefore, the ME bi–layer sensor alone would present a flaw in identifying the direction of magnetic field.

To mitigate this concern, the natural coupling between FM and AFM characteristic producing the exchange bias effect was applied to the ME sensor. PtMn and Cr_2_O_3_ were selected as the AFM materials to be coupled with Terfenol–D, the FM material. The exchange bias effect was simulated using MuMax^3^ simulation and observed through the FM hysteresis loop. The demonstration of the ME sensor as the hard disk drive (HDD) read head sensor was assumed; therefore, the FM/AFM—was set up with the maximum width of 36 nm, double the bit length at 2 Tb/in^2^ [11]. The structure length varied between 938, 400, and 200 nm, conforming with the optimal operating frequencies of 1.28 GHz, 3 GHz, and 6 GHz, respectively, of the current commercial TMR hard disk drives. Note that the first is the typical operating frequency while the latter two are the maximum operating frequencies of SATA II and SATA III standards, respectively [32]. Note also that the optimal frequencies satisfy *fl* = 1200 m/s. Since the force interaction is symmetrical due to Newton’s third law, the saturation magnetization in the AFM layer was deemed equal to that of the FM layer [24]. In addition, since the exchange stiffness of Cr_2_O_3_ was not found in the literature, they were thus assumed equal to other anti-ferromagnets such as IrMn and FeMn [24].

#### 4.2.1. Exchange Bias Characteristics of the Terfenol–D/PtMn Structure

**Figure 8:**
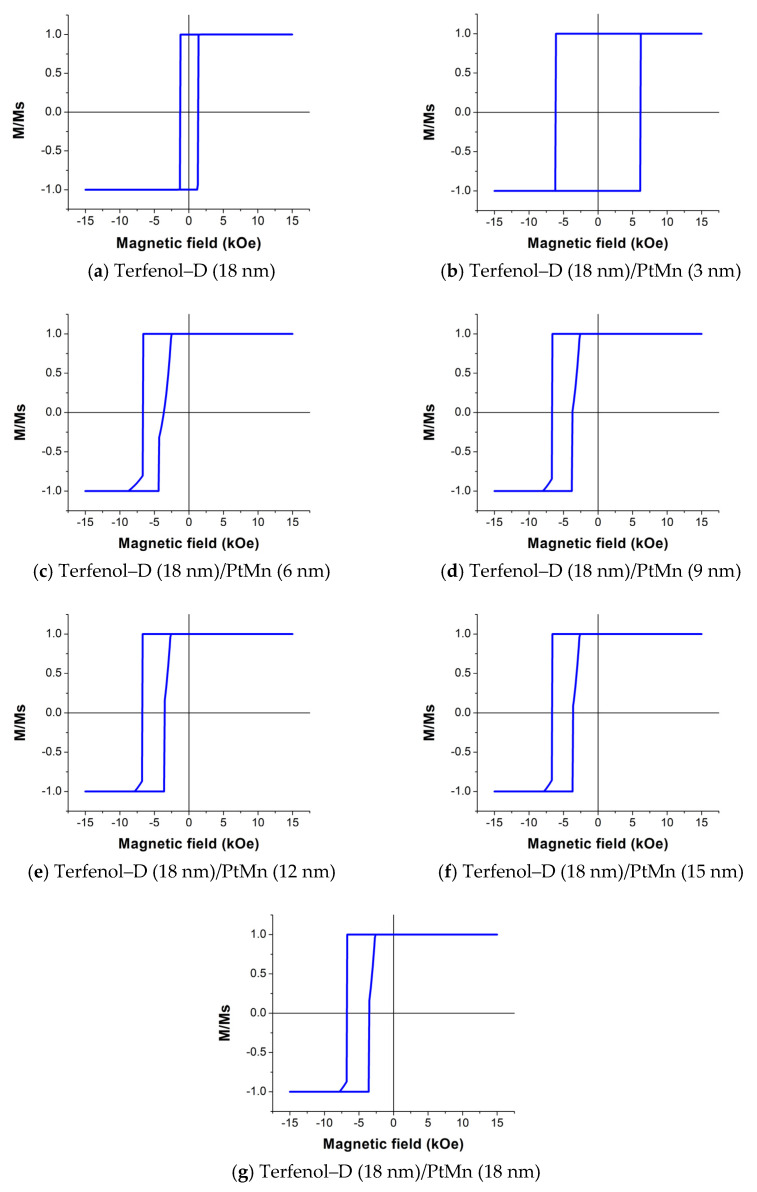
The hysteresis loops of 18 nm thick Terfenol–D coupling with PtMn at the PtMn thickness of (**a**) 0 nm, (**b**) 3 nm, (**c**) 6 nm, (**d**) 9 nm, (**e**) 12 nm, (**f**) 15 nm, and (**g**) 18 nm.

The hysteresis loops of the Terfenol–D with various PtMn thicknesses at *fl* = 1200 m/s are shown in Figure 8. Note that M/Ms refers to the ratio of the magnetization and magnetization saturation of Terfenol–D.

We found that the single Terfenol–D loop shown in Figure 8a exhibits relatively similar shape and coercivity to the experimental data reported previously [25,33]. A slight loop shift to the left is observed at the PtMn thickness of 6 nm and above while the loop widths are approximately the same. Although the hysteresis loop at 3 nm of PtMn is not shifted, the loop is wider than that at other thicknesses. Note that the plots in Figure 8 are the same for all three structure lengths, which thus implies that the structure length does not affect the exchange bias as long as the *fl* value is constant.

According to [34,35], the exchange bias characteristics of the AFM in the AFM/FM bi–layer can be explained by the *R*-ratio, defined in Mielkejohn and Bean’s (M–B) model as

(27) $$R=\frac{K_{AF}t_{AF}}{J_{eb}},$$

where $K_{AF}$ and $t_{AF}$ are the anisotropy constant and the thickness of the AFM, respectively, and $J_{eb}$ is the interface exchange energy.

At R≥1, the exchange bias field is finite, and the loop is shifted away from the y-axis. The magnetic moments of the AFM rotate reversibly following the complete reversal of the FM magnetic moments.

At 0.5≤R<1, the magnetic moments of the AFM follow those of the FM, but they are no longer reversible. The coercive fields are not shifted and the exchange bias field becomes zero. The loop exhibits the hysteresis–like behavior of the AFM.

At R<0.5, the magnetic moments of the AFM follow reversibly the magnetic moments of the FM. Therefore, the exchange bias field is zero and the coercive field increases with *R* and becomes finite. The hysteresis loop of the AFM–FM bilayer is quite similar to that of the FM.

**Figure 9:**
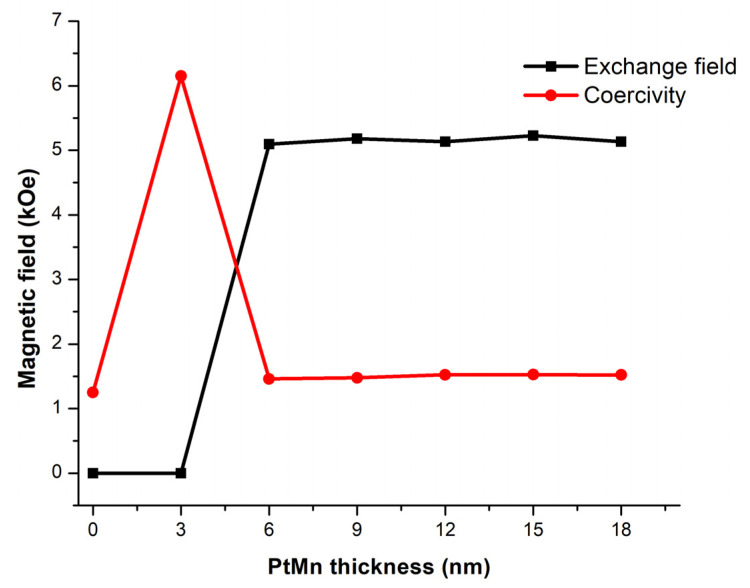
The plot of the exchange field and coercivity field of Terfenol–D coupling with PtMn at the PtMn thickness of 0–18 nm.

**Figure 10:**
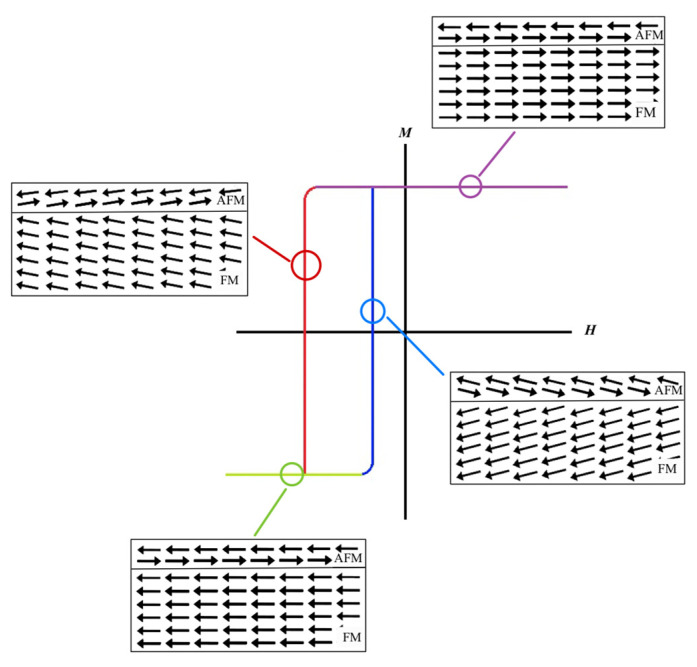
The magnetic moment of PtMn (AFM) and Terfenol–D (FM) associated with different locations on the Terfenol–D hysteresis loop. Note that *M* represents the magnetization of the FM material.

The plots of the exchange field and coercivity field of the PtMn/Terfenol–D structure shown in Figure 9 exhibit a sharp rise and drop at a PtMn thickness of around 6 nm or *R* = 1.39, which satisfies the exchange bias condition in Equation (27). Both fields reach equilibrium at approximately 5100 and 1500 Oe, respectively, also at the same thickness. Therefore, there is no additional advantage for thicker PtMn once the interfacial coupling effects are present. The magnetic moments associated with the locations on the hysteresis loop are illustrated in Figure 10.

#### 4.2.2. Exchange Bias Characteristics of the Terfenol–D/Cr_2_O_3_ Structure

**Figure 11:**
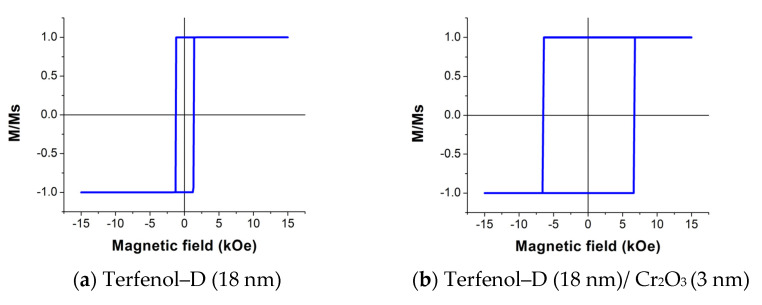

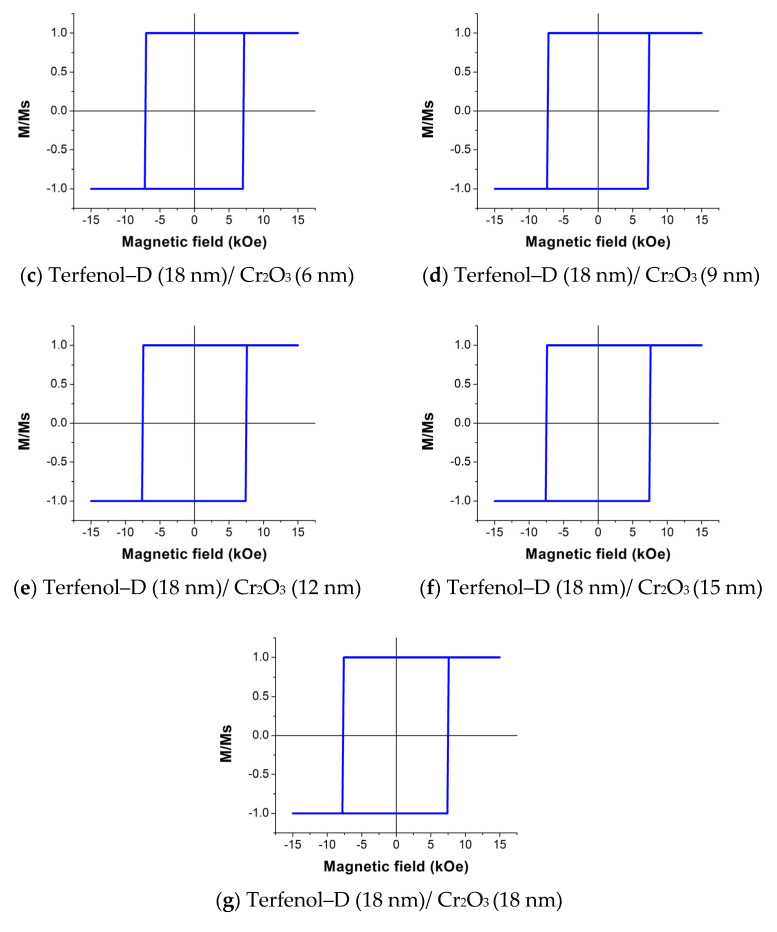
The hysteresis loops of 18 nm thick Terfenol–D coupling with Cr_2_O_3_ at the Cr_2_O_3_ thickness of (**a**) 0 nm, (**b**) 3 nm, (**c**) 6 nm, (**d**) 9 nm, (**e**) 12 nm, (**f**) 15 nm, and (**g**) 18 nm.

The hysteresis loops of the Terfenol–D with different Cr_2_O_3_ thicknesses at *fl* = 1200 m/s are shown in Figure 11. The results indicate that Cr_2_O_3_ could not induce the exchange bias phenomenon since no shift was observed. This is from Equation (27), since *K_AF_* of Cr_2_O_3_ is around 10 times smaller than that of PtMn, while its $J_{eb}$ is only 1.8 times smaller; therefore, the *R*-ratio is less than one for all Cr_2_O_3_ thicknesses. Adding Cr_2_O_3_ thickness in the range of interest only widens the hysteresis loop or increases the coercivity of the Terfenol–D. The results also indicate that the length is insignificant to the exchange bias effect as long as *fl* is constant.

**Figure 12:**
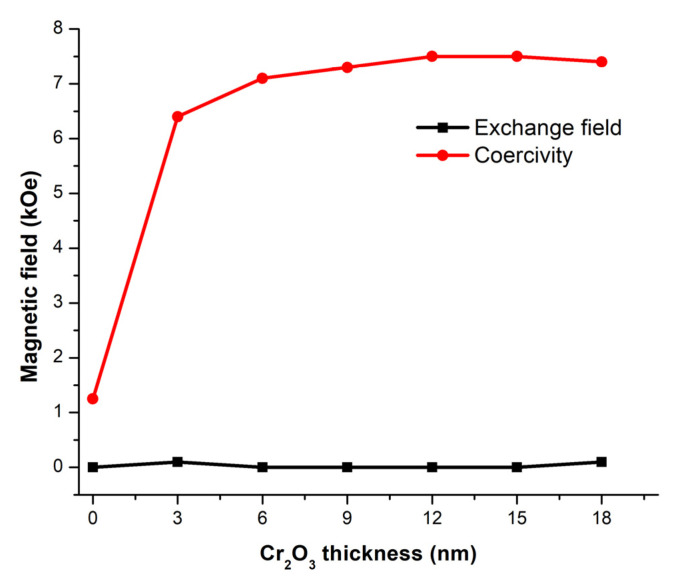
The plot of the exchange field and coercivity field of Terfenol–D coupling with Cr_2_O_3_ at the Cr_2_O_3_ thickness of 0–18 nm.

**Figure 13:**
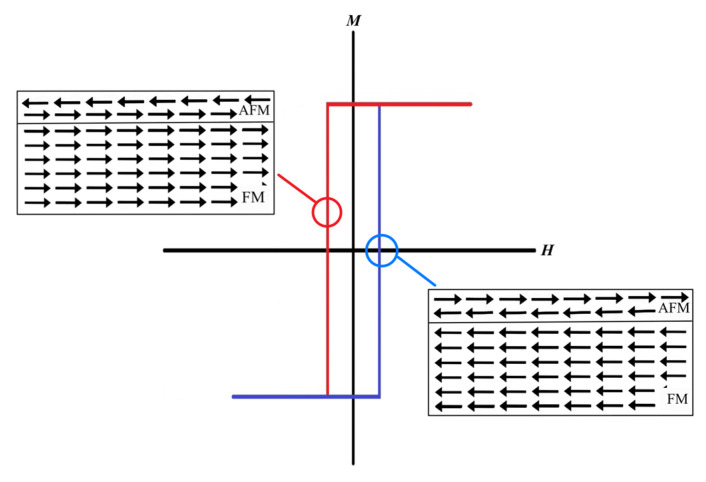
The magnetic moment of the Cr_2_O_3_ (AFM) and Terfenol–D (FM) associated with different locations on the Terfenol–D hysteresis loop. Note that, *M* represents the magnetization of the FM material.

Figure 12 shows the plot of the exchange field and coercivity field of the Cr_2_O_3_/Terfenol–D structure. The maximum coercivity is around 7500 Oe at 18 nm thick of Cr_2_O_3_. The magnetic moments associated with the locations on the hysteresis loop are illustrated in Figure 13.

### 4.3. The Demonstration of the Nano Tri–Layer PtMn/Terfenol–D/PZT Sensor

The demonstration of the nano tri-layer ME sensor consisting of the bi–layer Terfenol–D/PZT and PtMn in sensing magnetic field strength from a magnetic bit of FePt nanoparticles was conducted. Note that FePt is a high anisotropy magnetic material used as the medium of modern HDD. Our assumptions were as follows: (1) the size of a media disk was 3.5; (2) the areal density was 2 Tb/in^2^, which provided a field strength of around 7444 Oe [36]; (3) the lines of magnetic field from the magnetic bit were closed on themselves, independent from other bits; (4) the maximum of read spacing was 5 nm, smaller than that of 1 Tb/in^2^ [37]; and (5) the sensor was shielded magnetically following the design of the reported ME read head structure [10].

Since the maximum ME coefficient at *fl* = 1200 m/s resulted in the optimal thickness ratio of 0.19, at the Terfenol–D thickness of 18 nm the PZT thickness was then equal to 4.22 nm. The 6 nm thick PtMn was chosen here since the material effectively induced the exchange bias field for the tested structure. The occurrence of the exchange field at 5100 Oe from 6 nm PtMn thick made it possible to determine the polarity of the applied magnetic field.

**Figure 14:**
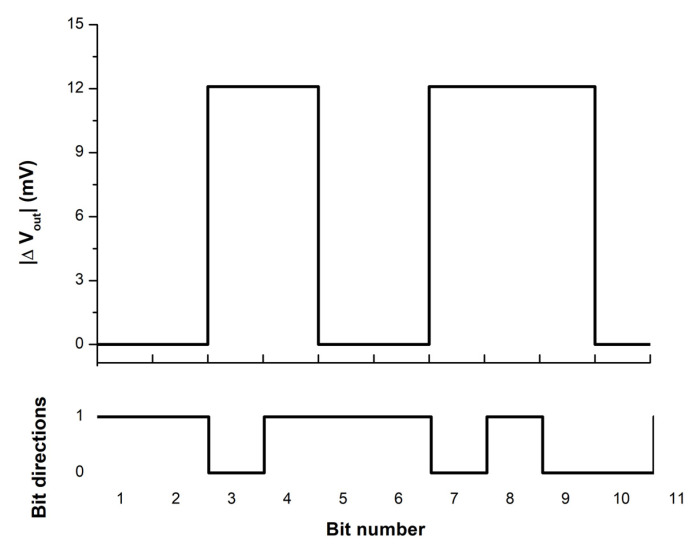
The different readback voltage for different magnetic bit directions.

The difference in output voltage associated with up and down magnetic fields was calculated from the average exchange field using Equation (26), and the result was 12.96 mV. This value is sufficient for magnetic field detection in most nanoscale magnetic sensing applications [38]. Figure 14 shows an example of the absolute output voltage difference (|∆*V_out_*|) of the demonstrated tri-layer ME sensor.

## 5. Conclusions

The geometry-dependent ME and EB effects of the tri–layer sensor consisting of the bi–layer multiferroic composites and AFM were investigated in this paper. The ME coefficient mathematical model of the L–T mode bi–layer bar structure was fully developed. It was shown that the ME coefficient depends largely on the structure, dimension, and operating frequency. The Terfenol–D/PZT bi–layer were chosen to demonstrate the ME effect. The ME coefficient of the bi-layer was computed at different thickness ratios and different *fl* values. It was shown that all ME coefficient profiles were bell-like shapes. The optimal thickness ratio decreased exponentially and its corresponding peaks ME coefficients increased exponentially with *fl*. The minimum and maximum peak ME coefficients were approximately 1756 and 5617 mV/Oe·cm at *fl* = 0.1 and 1200 m/s, respectively, which occurred at η = 0.43 and 0.19, respectively.

However, using the ME bi–layer alone presented a flaw in identifying the magnetic fields applied in opposite directions. The voltage outputs were of the same magnitude regardless of the direction. The EB effect from the coupling between Terfenol–D and PtMn and between Terfenol–D and Cr_2_O_3_ were simulated to observe and compare the exchange fields through the hysteresis loops of Terfenol–D using the Mumax^3^ simulation program. The exchange field was observed on the coupling between Terfenol–D and PtMn and occurred at the minimum PtMn thickness of 6 nm. The effect of the structure length was, however, not significant as long as *fl* was constant. Although the exchange field did not appear at the smaller thickness, an increase in the coercivity field did. The exchange and coercivity fields reached equilibrium at around 5100 and 1500 Oe, respectively. On the other hand, the bias field was not observed for the coupling between Terfenol–D and Cr_2_O_3_ due to the low anisotropy constant of Cr_2_O_3_ and the thickness range of interest. However, an increase in Cr_2_O_3_ thickness did increase the coercivity field. The maximum field was around 7500 Oe at 18 nm thick. The results from both PtMn and Cr_2_O_3_ proved that the EB effect significantly depends on types and the geometry of the structure.

The capability of the tri–layer ME sensor consisting of the PZT/Terfenol–D/PtMn was demonstrated in sensing the magnetic field strength from a magnetic bit of FePt at 2 Tb/in^2^ areal density. Since the optimal thickness ratio was around 0.19, Terfenol–D and PZT were chosen at 18 nm and 4.22 nm, respectively. The PtMn thickness was selected at 6 nm. For the demonstrated structure, the average exchange field was around 5100 Oe from 6 nm thick PtMn; therefore, the offset of the hysteresis loop can be used to determine the polarity of the applied magnetic field. The voltage output difference associated with the average exchange field of 5100 Oe was equal to 12.96 mV, which is sufficient to distinguish the direction of magnetic field and for detection in most nanoscale magnetic sensing technologies.

## Figures and Tables

**Table 1:** The properties of PZT and Terfenol–D.

Material	Density [kg/m^3^]	Elastic Compliance [m^2^/N]	Magnetostrictive Constant [m/A]	Piezoelectric Constant [m/V]	Electric Permittivity
Terfenol–D [22]	7700	10.7 × 10^−12^	11 × 10^−9^	n/a	n/a
PZT [23]	9250	17.9 × 10^−12^	n/a	60 × 10^−12^	617 $\varepsilon_{ο}$

**Table 2:** Terfenol–D, Cr_2_O_3_, and PtMn properties.

	Terfenol–D	Cr_2_O_3_	PtMn	Unit
Saturation magnetization	700 × 10^3^ [25]	0	0	A/m
Exchange stiffness	9 × 10^−12^ [26]	4 × 10^−12^ [24]	7.5 × 10^−12^ [27]	J/m
Anisotropy constant (in-plane)	n/a	2.00 × 10^5^ [28]	1.39 × 10^6^ [29]	J/m^3^

## Data Availability

The study did not report any data.
